# Inhalable Bottlebrush Polymer Bioconjugates as Vectors for Efficient Pulmonary Delivery of Oligonucleotides

**DOI:** 10.1021/acsnano.3c08660

**Published:** 2023-12-26

**Authors:** Yang Fang, Jiansong Cai, Mengqi Ren, Tongtong Zhong, Dali Wang, Ke Zhang

**Affiliations:** Department of Chemistry and Chemical Biology, Northeastern University, Boston, Massachusetts 02115, United States

**Keywords:** pulmonary delivery, antisense oligonucleotide, bottlebrush polymer, non-small-cell lung carcinoma, splicing correction

## Abstract

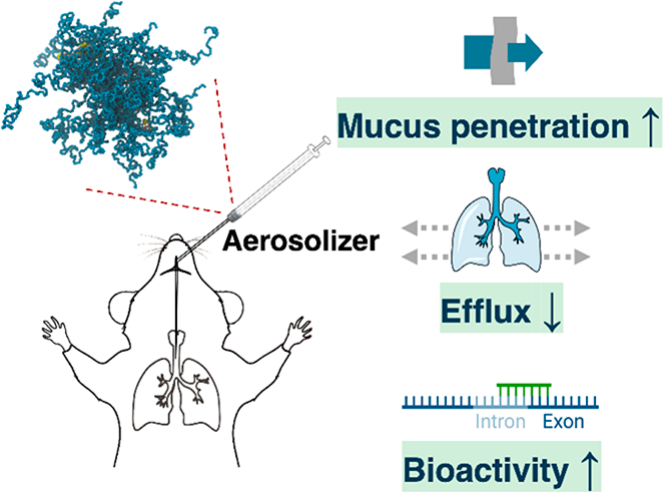

Antisense oligonucleotides hold therapeutic promise for various lung disorders, but their efficacy is limited by suboptimal delivery. To address this challenge, we explored the use of inhaled bottlebrush polymer–DNA conjugates, named pacDNA, as a delivery strategy. Inhaled pacDNA exhibits superior mucus penetration, achieving a uniform and sustained lung distribution in mice. Targeting the 5′ splice site of an aberrant enhanced green fluorescence protein (*EGFP*) pre-mRNA in EGFP-654 mice, inhaled pacDNA more efficiently corrects splicing than a B-peptide conjugate and restores EGFP expression in the lung. Additionally, in an orthotopic NCI-H358 non-small-cell lung tumor mouse model, inhaled pacDNA targeting wild-type *KRAS* mRNA effectively suppresses KRAS expression and inhibits lung tumor growth, requiring a substantially lower dosage compared to intravenously injected pacDNA. These findings demonstrate the potential of bottlebrush polymer–DNA conjugates as a promising agent for enhanced oligonucleotide therapy in the lung and advancing the treatment landscape for lung disorders.

## Introduction

The inhalation route of administering therapeutics is a promising approach to treat pulmonary diseases, as it leads to the direct delivery of drugs to the site of pathology, reducing the risk of adverse side effects on healthy organs and tissues.^1,2^ In addition, inhaling drugs provides a high local concentration, enhancing efficacy with lower doses.^3,4^ The delivery of nucleic acid therapeutics such as antisense oligonucleotides (ASOs) and small interfering RNAs (siRNAs) via inhalation has been evaluated for the treatment of various lung diseases including lung cancer, cystic fibrosis (CF),^5^ asthma,^6^ and chronic obstructive pulmonary disease (COPD).^7^ Despite the potential benefits, the delivery of nucleic acids via inhalation has not yet shown clinically significant benefits, likely due to insufficient gene transfer to the target site.^8^

A main barrier to effective delivery of antisense oligonucleotides to the lung is the normal airway mucus, which overlies the airway epithelium and reduces gene transfer efficiency.^9,10^ Nonviral nucleic acid delivery vehicles are typically polycationic, which facilitates nucleic acid complexation and protection, cell uptake, and endosomal disruption.^11^ However, such cationic gene transfer agents are often trapped in the airway mucus gel layer due to electrostatic interactions and are rapidly removed from the lung by mucociliary clearance or cough-driven clearance, resulting in insufficient delivery.^12,13^ In addition, the airway mucus from patients with lung disorders such as CF and COPD often has tighter mesh structure, reduced average pore size (140 ± 50 nm),^14^ and increased viscoelasticity and thickness compared to normal respiratory mucus, adding to the challenge for nanocarriers to penetrate the mucus barrier.^15^ To overcome this obstacle, inhaled nanocarriers must be small enough to diffuse through the mucus and have a mucus-inert surface to avoid adhesion with mucus components.^16^ For example, a dense coating of the uncharged polymer, poly(ethylene glycol) (PEG), over the nanoparticle has been demonstrated to improve diffusion through mucus by decreasing mucoadhesion.^17−21^

Recently, our group has developed a high-density PEG–ASO conjugate termed pacDNA (polymer-assisted compaction of DNA), which consists of ∼2 antisense oligonucleotides covalently tethered to the backbone of a bottlebrush polymer with ∼30 PEG_10k_ side chains, resulting in a much denser PEG coverage than typical PEGylated pharmaceuticals.^22^ The pacDNA retains the ability to hybridize to the complementary target sequence without releasing the ASO from the polymer, despite the dense polymer coverage. The pacDNA is hydrodynamically ∼30 nm and is nearly charge-neutral (with a slight negative charge), which makes it ideal for mucus penetration. Importantly, the pacDNA is a safe, single-entity agent that can transfect cells efficiently without an added co-carrier, which often leads to toxic and immunological difficulties. Prior safety analysis of the pacDNA revealed no acute toxicity and minimal antipolymer immunogenic response after repeated intravenous (i.v.) dosing.^22^ Here, we explore the potential of pacDNA to improve delivery of oligonucleotide to the lung through local inhalation administration. We evaluated the delivery efficiency of an inhaled pacDNA containing a splice-switching oligonucleotide (SSO) using enhanced green fluorescence protein (EGFP)-654 transgenic mice, which express an aberrantly spliced EGFP-654 pre-mRNA reporter ubiquitously.^23^ Furthermore, we assessed the gene regulation efficiency and antitumor efficacy in an orthotopic mouse model of non-small-cell lung carcinoma (NSCLC) bearing KRAS mutation.

## Results and Discussion

### Preparation and Characterization of pacDNA

**Figure 1:**
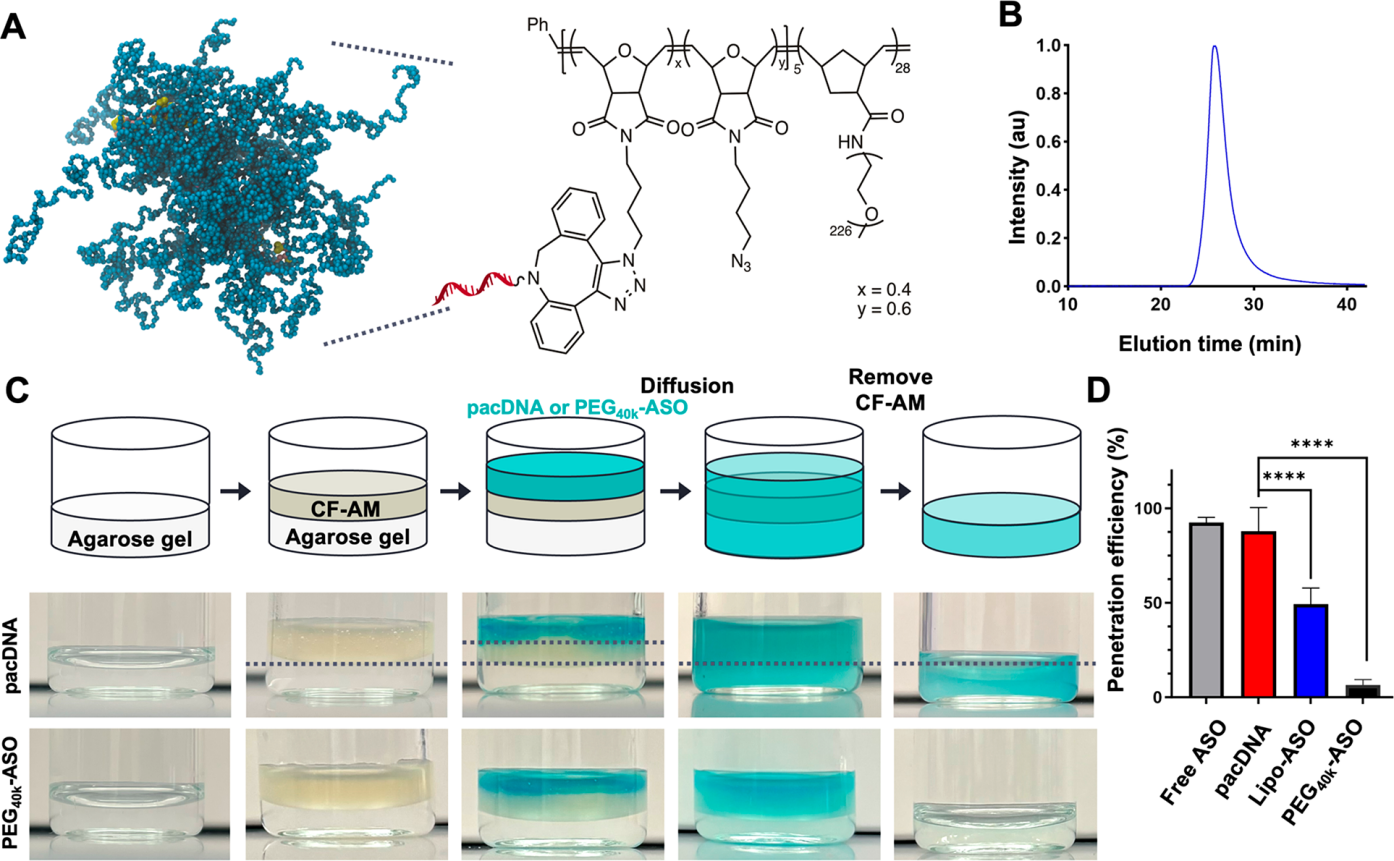
Structure of pacDNA and mucus penetration. (A) Simulated (molecular dynamics) and chemical structures of pacDNA. (B) Aqueous gel-permeation chromatogram of pacDNA, showing monomodal distribution and low polydispersity. (C) Facile penetration of pacDNA across cystic fibrosis artificial mucus layer (CF-AM) compared to the PEG_40k_-ASO control group. (D) Extent of mucus penetration after 24 h of incubation at 37 °C. ****p < 0.0001 (two-tailed test).

The bottlebrush polymer and the pacDNA used in this study were synthesized according to prior literature (Scheme S1).^24−28^ The ASO sequences tested are listed in Table S1. Briefly, the diblock bottlebrush polymer was prepared via sequential ring-opening metathesis polymerization (ROMP) of two monomers, a 7-oxanorbornenyl bromide (NBr)^29^ and norbornenyl-modified PEG (NPEG) at a ratio of 5:35, which gave a diblock architecture (pNBr_5_-*b*-pNPEG_28_) with an *M*_n_ ∼ 280 kDa, as characterized by *N,N*-dimethylformamide gel permeation chromatography (DMF-GPC, Figure S1A). Following azide substitution and subsequent coupling with dibenzocyclooctyne (DBCO)-modified ASO strands via copper-free click chemistry, pacDNA structures with an average of two ASO strands per polymer were synthesized (Figure 1A). The conjugates were purified by aqueous size-exclusion chromatography (Figure 1B). Successful synthesis and purification of pacDNA was confirmed by gel electrophoresis (Figure S1B). Dynamic light scattering shows the presence of nanoparticles with a hydrodynamic diameter (Z-average) of 29 ± 3 nm, and zeta potential measurements indicate that the pacDNA (in unbuffered Nanopure water, pH 6.4) has a slight negative charge of −5 to −3 mV, which has significantly reduced negative surface charge compared to free ASO of ∼−37 mV (Figure S1C).

### Mucus Penetration

To study the extent of mucus penetration, we employed an assay utilizing cystic fibrosis artificial mucus (CF-AM), as described in previous studies.^30^ Briefly, Cy5-labeled pacDNA and controls (free ASO, Lipofectamine-complexed ASO, and linear PEG_40k_-ASO) was applied to the top of the CF-AM layer, which had been previously placed in a vial. An agarose gel was positioned beneath the CF-AM layer to capture pacDNA molecules that diffused through the CF-AM. The vial was then incubated at 37 °C for 24 h. After the incubation, the CF-AM containing pacDNA was removed, and the remaining gel was rinsed, collected, and melted at 60 °C, allowing for the quantification of the extent of penetration (Figure 1C). The penetration for pacDNA was measured to be 87.9%, which is significantly greater compared with the cationic lipid vector-complexed control (49.2%) and PEG_40k_-ASO (6.4%) (Figure 1D). The facile penetration is attributed to the charge-neutral and nonhydrophobic nature of the pacDNA, which allows it to evade the anionic mucus and pulmonary surfactants. While free ASO is also able to penetrate the mucus (92.5%), its bioactivity is expected to be limited if unaided by a transfection agent. We attribute the inability of PEG_40k_-ASO to penetrate the mucus to the chain entanglement of long linear PEG with the mucus mesh. Bottlebrush polymers, on the other hand, exhibit a denser, more globular conformation,^31^ which reduces intermolecular entanglement, allowing for more facile diffusion through the mucus network. These data suggest that the pacDNA operates as a mucus-inert material, being able to penetrate the mucus layer and gain access to the underlying epithelium.

### Biodistribution and Pharmacokinetics

**Figure 2:**
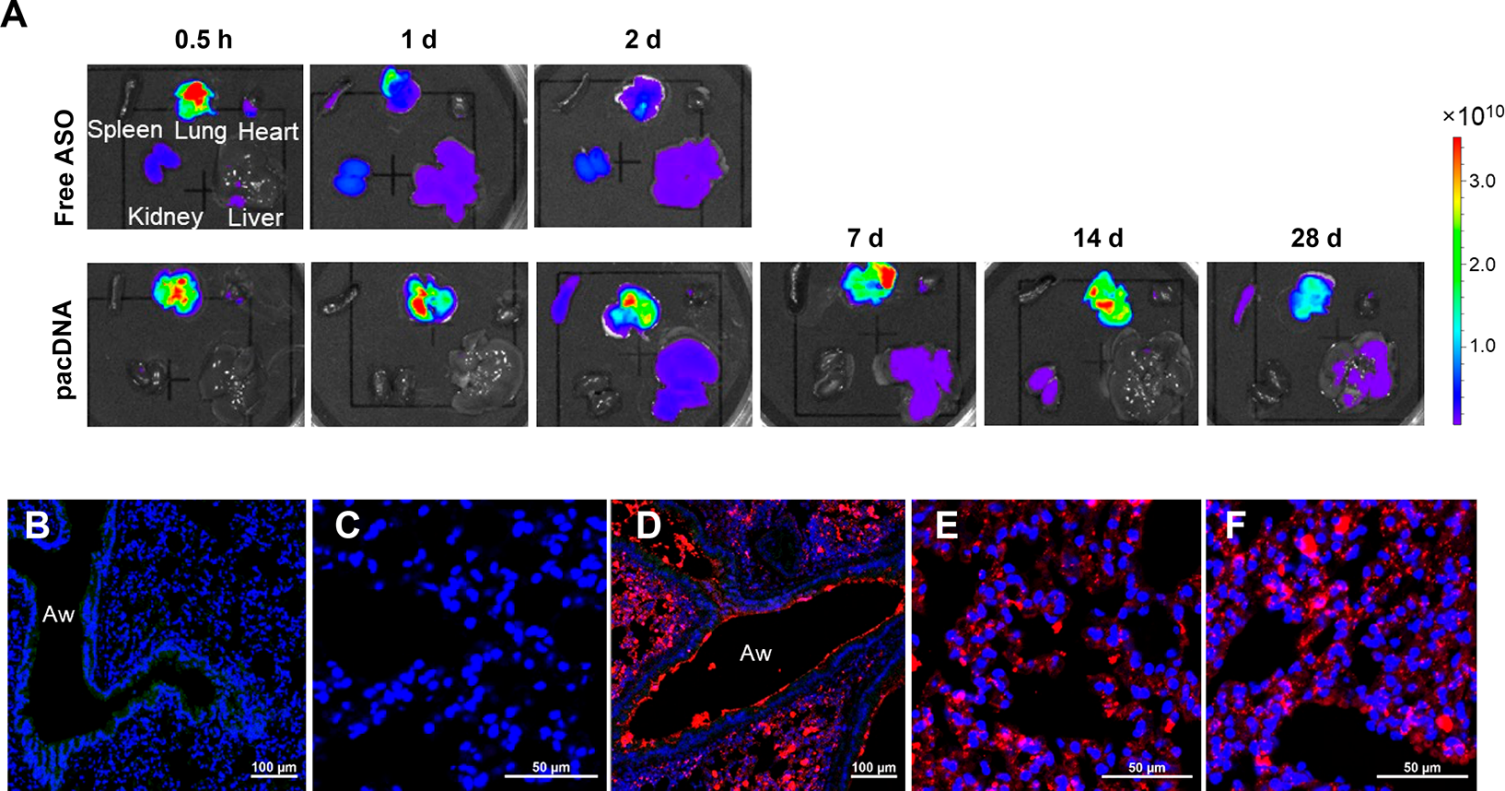
The distribution of pacDNA in normal mouse lung (C57BL/6). (A) *Ex vivo* fluorescence imaging of lung and other major organs at various time points postinhalation. (B–F) Representative images of pacDNA distribution in the mouse lung airway and parenchyma following intratracheal microsprayer administration. (B, C) Untreated lung airway and parenchyma. (D, E) Distribution of Cy5-labeled pacDNA (red) in the airway and alveolar region 24 h postinhalation. (F) Distribution of pacDNA 7 d postinhalation. Cell nuclei are stained with Hoechst 33342 (blue), Aw (lung airway), and autofluorescence (green).

To explore the accumulation of inhaled pacDNA in the mouse lung and its biodistribution *in vivo*, aerosolized Cy5-labeled free oligonucleotide (with full locked nucleic acid, or LNA, modification) and its bottlebrush formulation (pacDNA_LNA_) were delivered to C57BL/6 mouse lung intratracheally via a microsprayer.^32^ Animals were sacrificed at various time points after sample administration. Fluorescence imaging of dissected organs showed that the free LNA achieves a high lung concentration in the first 30 min, which falls off rapidly to ∼20% within the first 24 h. In contrast, the pacDNA is retained in the mouse lung for at least 4 weeks postinhalation, with no obvious signal decreases for the first 2 weeks (Figure 2A). In addition, administration of the free LNA resulted in detectable signals in the kidney within the first 30 min, suggesting that some LNA (or the free dye metabolite) has made its way into the bloodstream, which leads to rapid renal clearance. In contrast, no kidney accumulation was observed for pacDNA. Both free LNA and pacDNA exhibited some accumulation in the liver.

To further understand the distribution of pacDNA within the mouse lung, fluorescence imaging of cryosectioned lung tissues at different time points postinhalation of pacDNA or a low-PEG density control, PEG_40k_-ASO, was performed. Signals of pacDNA were observed not only from the epithelium of the airways but also throughout lung parenchyma. Uniform distribution of pacDNA was observed in the lung airway and alveolar region at different time points postinhalation (Figure 2B–F).^33−35^ In contrast, signals for the linear PEG_40k_-ASO were more sparsely distributed in the lung (Figure S2), which may be due to insufficient mucus penetration. Plasma pharmacokinetics of inhaled pacDNA and free LNA was also assessed for up to 14 days. The pacDNA exhibits significantly greater plasma concentration compared with free LNA throughout the test period. The plasma concentration of pacDNA at 14 days postinhalation was similar to that of free LNA at 4 h postinhalation (Figure S3). These results suggest faster penetration of the free LNA into the bloodstream compared to pacDNA, leading to more rapid clearance and shorter retention in the lung.

### Splicing Correction Using the EGFP-654 Mouse Model

**Figure 3:**
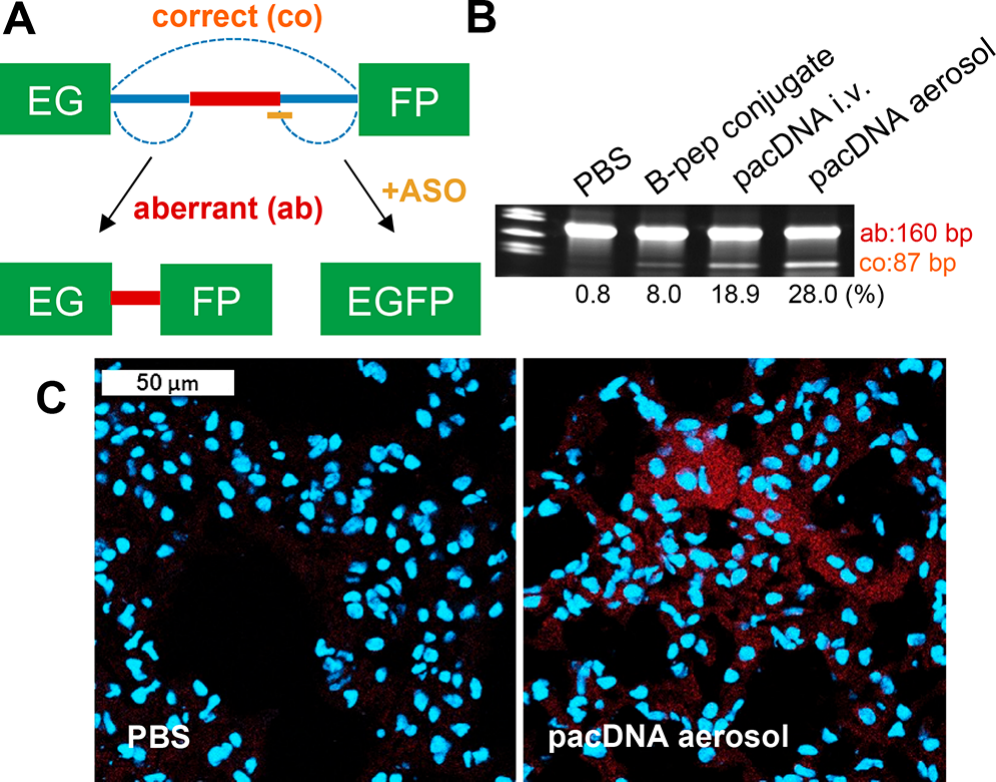
Splicing correction efficacy study of inhaled pacDNA using EGFP-654 mice. (A) EGFP-654 transgenic mice with a modified EGFP-654 gene, wherein the coding region of the EGFP (green boxes) is interrupted by an aberrantly spliced intron from the human β-globin IVS2-654. This leads to retention of an intron fragment (red bar) in the mature mRNA that prevents EGFP translation. By blocking the aberrant 5′ splice site with SSO-654 (yellow bar), correct splicing is restored. (B) RT-PCR of EGFP RNA from the lung tissue of EGFP-654 mice. Bands from aberrantly spliced and correctly spliced EGFP mRNA are shown as “Ab” (160 bp) and “Co” (87 bp), respectively. (C) Immunofluorescence-stained lung cryosections of mice treated with PBS or inhaled pacDNA-654. Cell nuclei was stained with Hoechst 33342 (blue). EGFP was not directly detected due to autofluorescence. Instead, anti-GFP antibody Alexa Fluor 594 (red) was used.

To evaluate the antisense effect of inhaled pacDNA and compare its efficacy to i.v. delivered pacDNA *in vivo*, EGFP-654 transgenic mice, which ubiquitously express a modified EGFP pre-mRNA containing an aberrantly spliced human β-globin intron (IVS2-654), were used.^36^ Delivery of an SSO (SSO-654) specifically targeting the aberrant 5′ splice site in EGFP-654 pre-mRNA (Figure 3A) leads to correction of the aberrant splicing and subsequent production of correct EGFP.^23^ Mice were equally divided into six groups to receive inhaled phosphate-buffered saline (PBS), inhaled pacDNA-654 (3 nmol, SSO basis), inhaled scrambled pacDNA-654 (pacDNA-654 Scr, 3 nmol, SSO basis), inhaled brush polymer only (equal amount to pacDNA groups), i.v. delivered pacDNA-654 (20 nmol, SSO basis), and i.v. delivered B peptide–SSO conjugate (an arginine-rich cell-penetrating peptide, 20 nmol, SSO basis).^37^ All samples were given to the animals once a day for four consecutive days (Figure S4), and animals were sacrificed 1 week after the last administration. Reverse transcriptase-polymerase chain reaction (RT-PCR) analysis shows that inhaled pacDNA-654 restored splicing more efficiently than other test groups, despite being given at only 15% that of the i.v. dosage (Figures 3B, S5). Inhaled pacDNA also successfully restored the EGFP protein expression in mouse lung, which was confirmed by immunofluorescence (IF) staining (Figure 3C). These results demonstrate that the pacDNA can effectively cross the mucus layer, enter the nucleus of lung cells, and engage with its pre-mRNA target.

### Orthotopic Mouse Model for *KRAS*^mut^ Non-Small-Cell Lung Carcinoma

Next, we studied the efficacy of pacDNA targeting the human Kirsten rat sarcoma oncogene (*KRAS*) in an orthotopic NSCLC mouse model. An ASO sequence targeting the 3′ untranslated region (3′ UTR) of the *KRAS* mRNA was adopted. The targeted region is away from mutation sites; thus, wild-type KRAS is depleted, making this sequence potent against all mutant isoforms. Two ASO chemistries were used: unmodified phosphodiester (PO) chemistry and LNA modification. In addition, pacDNAs containing scrambled controls of each of the ASOs were prepared. We first tested the gene regulation efficiency in NCI-H358-Luc cells, a human KRAS^G12C^ NSCLC line stably transfected with luciferase. Cells were treated with pacDNAs and the controls for 72 h. The most effective gene regulation was observed for pacDNA_LNA_, which was able to reduce KRAS expression by 71% (10 μM, Figure S6), which compares favorably with pacDNA_PO_ (60%). In contrast, the scrambled controls did not result in appreciable downregulation. A 3-(4,5-dimethylthiazol-2-yl)-2,5-diphenyl tetrazolium bromide cytotoxicity assay indicated dose-dependent antiproliferation activity for pacDNA_LNA_, while the brush polymer and scrambled pacDNA_LNA_ exhibited nearly no cytotoxicity (Figure S7). Given these studies, the pacDNA_LNA_ was selected for subsequent antitumor *in vivo* studies.

**Figure 4:**
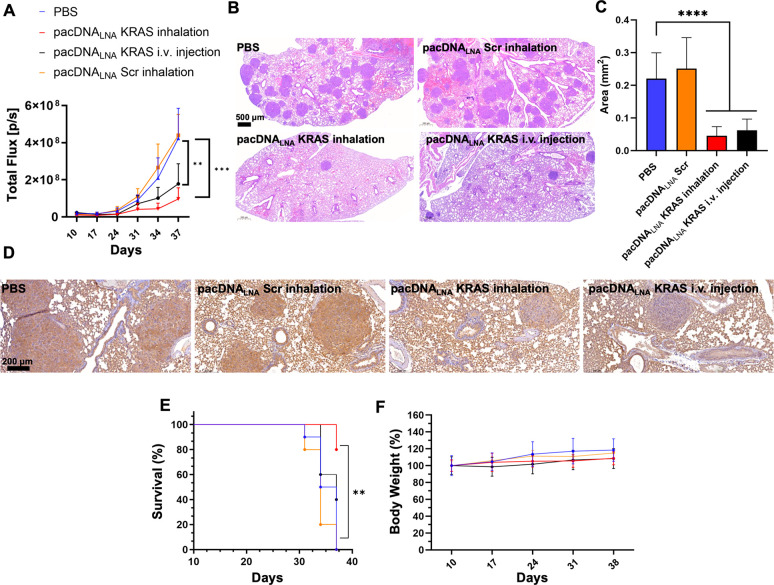
Antitumor efficacy of inhaled pacDNA using NCI-H358 orthotopic lung tumor mice. (A) Anticancer efficacy of inhaled or i.v. delivered pacDNA_LNA_ in NCI-H358 orthotopic lung tumor mouse model. Statistical analysis was performed using one-way ANOVA followed by Dunnett’s *post hoc* test. **p < 0.01, ***p < 0.001. (B) Representative hematoxylin and eosin (H&E) staining of day 38- harvest lung tissue from different treatment groups. Scale bar: 500 μm. (C) The area of individual tumor nodules of each group was measured according to the H&E stain images. ****p < 0.0001 (two-tailed test). (D) Representative immunohistochemical staining for KRAS in day 38-harvest lung tissues, showing reduced KRAS expression of nodules in inhaled pacDNA_LNA_ KRAS-treated groups vs controls. Scale bar: 200 μm. (E) Kaplan–Meier survival curves for NCI-H358 orthotopic lung tumor mice in each treatment group. End point = 1.5 × 10^8^ p/s. (F) Body weight changes of NCI-H358 orthotopic lung tumor mice during the treatment period. For animal survival analysis, statistical significance was calculated by the log-rank test, **p < 0.01.

Next, we studied pacDNA_LNA_ using an orthotopic NSCLC mouse model, which was established using NCI-H358-Luc cells by inoculating the cells (5 × 10^5^) into the tail vein of the NOD SCID mouse. We first examined the distribution of inhaled pacDNA_LNA_ in the mouse lung. At 24 h postinhalation, mice were euthanized, and lung tissues were collected. Confocal microscopy of the cryosectioned lung revealed that the Cy5-labeled pacDNA_LNA_ was able to enter the mouse lung and penetrate tumor nodules (Figure S8). To study antitumor activity, we dosed tumor-bearing mice with pacDNA_LNA_ twice weekly, at a dosage of 0.15 μmol/kg for inhalation or 0.5 μmol/kg for i.v. injection (ASO basis) using the schedule depicted in Figure S9 and monitored tumor growth by bioluminescence imaging. Detected signal intensity can be linearly correlated with tumor growth, as photon emission exclusively originates from luciferase-expressing cells.^38,39^ At 10 days after tumor cell inoculation, mice showing comparable initial bioluminescence intensities were separated into four groups to receive pacDNA_LNA_ (inhalation), pacDNA_LNA_ (i.v.), scrambled pacDNA_LNA_ (inhalation), and PBS (inhalation). Both pulmonary delivery and i.v. delivery of pacDNA significantly repressed the growth of tumor cells compared to control groups (Figures 4A,S10). However, the inhalation delivery route resulted in better tumor growth suppression than systemic administration despite lower total dosage. Thirty-eight days after initial tumor cell inoculation, lungs from animals in each group were collected for imaging and histological analysis. The numbers of tumor nodules and their sizes were visibly reduced for treatment groups vs controls (Figure S11), which is corroborated by hematoxylin and eosin (H&E) staining (Figure 4B,C). KRAS expression levels were characterized by immunohistochemical staining (IHC) of the tumor cryosections, which shows that the inhaled pacDNA_LNA_ induced a marked reduction of KRAS levels in the tumor nodules compared to controls (Figures 4D, S12A,B), as indicated by a reduction of the brown deposits. Of note, the antibody used in the IHC staining cannot differentiate mouse and human KRAS; thus the non-nodule, normal lung tissues exhibit a background level of KRAS expression that is unaffected by the treatment (pacDNA_LNA_ targets only human *KRAS* mRNA). Kaplan–Meier survival analysis using log rank test for comparison between pacDNA_LNA_ KRAS (inhalation) and controls (end point: 1.5 × 10^8^ photons/second) revealed a significant difference in overall survival (Figure 4E). The body weights of the tumor-bearing mice, which were recorded once a week, did not show significant differences between the pacDNA-treated groups and the PBS group, suggesting that the pacDNA treatment is well tolerated in mice (Figure 4F).

## Study Limitations

Despite the impressive capability of pacDNA to penetrate mucus and enhance gene regulation in the lungs through inhalation administration with massively reduced dosage requirements in comparison to a clinical ASO,^40^ our study was confined to mice, whose KRAS mRNA sequence in the targeted region is not homologous to human KRAS. Therefore, potential side effects caused by downregulating wild-type KRAS systemically are not revealed in these studies. To evaluate these potential effects, nonhuman primates must be used, as their KRAS mRNA is identical to the human sequence in the targeted region.

Furthermore, our *in vivo* efficacy studies did not achieve stasis or regression of tumor growth, suggesting that KRAS depletion as a single-agent approach may be insufficient. The effect of inhibiting KRAS^mut^ for lung adenocarcinoma cells using trial drugs has been shown to be markedly improved by simultaneously inhibiting insulin-like growth factor 1 receptor (IGF1R) and mammalian target of rapamycin (mTOR).^41^ Since commercial drugs are available for both targets (brigatinib and rapamycin analogs, the former is already used for NSCLC), there is a solid basis to design combination treatments using two- or three-drug combinations. Combinations of EGFR inhibitors and KRAS depletion have also shown promising synergistic effects.^42^ The therapeutic efficacy of the current single-gene ASO strategy may also be improved by combining node-targeted ASOs such as KRAS + PIK3A/B.

## Conclusion

The lung offers an opportunity for therapeutic intervention, as it can be directly and specifically targeted by pulmonary delivery, which reduces the possibilities of systemic side effects and tolerability concerns. Oligonucleotides are a promising therapeutic modality for the treatment of pulmonary diseases, as they can inhibit pathways that are otherwise difficult to target. However, the mucus layer, pulmonary surfactants, and rapid mucociliary clearance and efflux from the lungs reduce the efficacy and durability of a potential oligonucleotide treatment. Conventional cationic polymeric, peptide, or lipid formulations that enhance the delivery of oligonucleotides face a difficult dilemma for pulmonary delivery: features that make them more potent *in vitro*, such as high charge density and lipophilicity, also make them interact more strongly with the anionic, gel-like mucus layer and the high lipid content of pulmonary surfactant, reducing their permeability and accelerating their clearance. A noncationic vector capable of penetrating the physiological barriers of the lung, increasing pulmonary retention, and enhancing transfection efficiency is still very much sought after.

Our studies have shown that the inhalation method of administering pacDNA, which can be considered as a densely PEGylated form of oligonucleotide, results in efficient mucus and pulmonary tissue penetration as well as prolonged retention in the lung, leading to efficient gene regulation using antisense oligonucleotides. The higher density of PEG found in pacDNA relative to conventional PEGylation using high molecular weight PEG allows it to effectively penetrate airway mucus, resulting in uniform distribution throughout the mouse lung airways and parenchyma. Inhalation of pacDNA targeting pre-mRNA of EGFP 654 mice efficiently corrects aberrant splicing with higher splice switching activity and reduced dosage compared to the i.v. delivery route and B peptide conjugate. Furthermore, inhaled pacDNA targeting wild-type KRAS shows superior efficacy for inhibiting tumor growth in an orthotopic mouse model of *KRAS*^mut^ NSCLC at a lower dose. Overall, these findings underscore the immense potential of pacDNA as a powerful vehicle for delivering oligonucleotides to the lung via inhalation, offering promising therapeutic prospects for various lung-related disorders.

## Materials and Methods

### Preparation of Cystic Fibrosis Artificial Mucus

A CF-AM formulation was prepared following established protocols described in previous literature.^30,43^ In brief, 500 mg of DNA, 250 μL of sterile egg yolk emulsion, 250 mg of mucin, 0.295 mg of diethylenetriamine pentaacetate (DTPA), 250 mg of casamino acid, 250 mg of NaCl, and 110 mg of KCl were mixed together in a final volume of 50 mL of sterile DNase-free water. The mixture was stirred thoroughly at RT for 2 h to achieve a homogeneous dispersion before use.

### Penetration of Cy5-Labeled pacDNA and Controls in CF-AM

The mucus-penetrating ability of pacDNA, PEG_40k_-ASO, free ASO, and Lipofectamine mixed with ASO (100 ± 2 nm, Z-average size; zeta potential +25 mV) was evaluated using a modified penetration test in CF-AM based on a previously described method.^44^ A 0.28 w/v % agarose solution was prepared in hot Nanopure water. Vials with a diameter of 23 mm were filled with 1 mL of the agarose solution, hardened at room temperature, and stored at 4 °C until further use. One milliliter of CF-AM was applied to the hardened agarose gel. A 200 μL amount of Cy5-labeled pacDNA solution (10 nmol dissolved in water, ASO basis), Cy5-labeled free ASO, Cy5-labeled PEG_40k_-ASO, or Cy5-labeled free ASO mixed with Lipofectamine 3000 based on the standard protocol was then added onto the CF-AM layer and incubated at 37 °C. After a 24 h incubation period, CF-AM containing pacDNA or controls was withdrawn, and the remaining agarose gels were rinsed with 1 mL of Nanopure water three times. The gels were subsequently melted at 60 °C, transferred to a 96-well black plate, and analyzed by measuring the fluorescence intensity of Cy5 using a BioTek Synergy Neo2 multimode microplate reader (BioTek Inc., VT, USA). The amount of Cy5-labeled pacDNA and controls that reached the agarose gel was measured against a standard curve generated using Cy5-labeled pacDNA, Cy5-labeled PEG_40k_-ASO, or Cy5-labeled free ASO dissolved in an agarose gel solution. The penetration efficiency is calculated as the ratio of the detected concentration of ASO in the gel vs the maximum possible concentration if the ASO were to freely diffuse. Therefore, if no Cy5 signal is detected in the gel, then the penetration efficiency is 0%. If the concentration of Cy5 in the gel is the same as that in the top layer (mucus), then the penetration efficiency is 100%.

### Animal Studies

All animal protocols were approved by the Institutional Animal Care and Use Committee of Northeastern University and carried out under pathogen-free conditions in the animal facility of Northeastern University and in accordance with the National Institutes of Health animal care guidelines. Female C57BL/6 mice (6–8 weeks old) were purchased from Charles River Laboratories, female NOD. Cg-*Prkdc*^scid^/J (NOD SCID) mice (6–8 weeks old) and Tg(CAG-EGFP*)1Rkol/RjulJ mice (Cryo Recovery) were purchased from The Jackson Laboratory. The animals were given free access to a standard laboratory diet and water and were kept in the laboratory animal facility with temperature and relative humidity maintained at 23 ± 2 °C and 50 ± 20%, respectively, under a 12 h:12 h light:dark cycle. Mice were given at least 1 week to acclimatize to the new environment and housing conditions of the animal facility prior to experiments.

### *Ex Vivo* Organ Imaging

Cy5-labeled pacDNA or free LNA (5 nmol dissolved in 50 μL of PBS, ASO basis) was administrated intratracheally to C57BL/6 mice using a microsprayer. At various time points postinhalation of free LNA (0.5 h, 1 d, 2 d) or pacDNA (0.5 h, 1 d, 2 d, 7 d, 14 d, 28 d), mice were sacrificed, and lungs and other major organs were harvested for fluorescence imaging using an IVIS Lumina II imaging system (Caliper Life Sciences, Inc., MA, USA).

### Fluorescence Microscopy of Sectioned Mouse Lung

Cy5-labeled pacDNA or Cy5-labeled PEG_40k_-ASO (5 nmol dissolved in 50 μL of PBS, ASO basis) was administrated intratracheally to C57BL/6 mice and orthotopic NCI-H358 lung tumor mice using a microsprayer. At 24 h and 7 d postadministration, mouse lung tissues were immediately frozen in OCT compound (Fisher Scientific Inc., USA), sectioned, stained with Hoechst 33342, and imaged on an LSM-700/LSM-800 confocal laser scanning microscope (Carl Zeiss Ltd., Cambridge, UK).

### RNA Isolation and Analysis of EGFP-654 Mouse Lung

Frozen mouse lung tissue (∼30 mg) was homogenized using a BeadBlaster 24R refrigerated homogenizer (Benchmark) at 3650 rpm for 30 s, with a linear speed of 6.00 m/s at 4 °C. Two cycles were performed at intervals of 30 s. Total RNA was extracted from the homogenized tissue using the RNeasy fibrous tissue mini kit (Qiagen) following the standard procedures. The EGFP mRNA was amplified by one-step RT-PCR using the SuperScript IV one-step RT-PCR system (Invitrogen) following the recommended protocol. In brief, 100 ng of isolated RNA was used for the RT-PCR reaction, which proceeded at 55 °C for 10 min and 98 °C for 2 min, followed by 40 cycles of amplification at 98 °C for 10 s, 68 °C for 10 s, and 72 °C for 15 s, and final extension at 72 °C for 5 min. The resulting PCR products were separated on a 4–20% gradient nondenaturing polyacrylamide gel, and bands were visualized by staining with GelRed nucleic acid gel stain in 0.1 M NaCl solution for 30 min (Biotium, Inc.) EGFP forward primer: 5′-CGTAAACGGCCACAAGTTCAGCG-3′, reverse primer: 5′-GTGGTGCAGATGAACTTCAGGGTC-3′.

### Immunofluorescence Staining of EGFP Mouse Lung

For immunofluorescence analysis, cryostat-cut sections of lungs of PBS-treated mice and inhaled pacDNA-654-treated mice were fixed in 4% paraformaldehyde for 20 min and blocked with 2.5% normal goat serum for 30 min. Sections were then stained with anti-GFP antibody at 1:200 dilution (GFP polyclonal antibody Alexa Fluor 594, catalog no. A-21312; Thermo Fisher Scientific). Samples were stained with Hoechst 33342 for 10 min and imaged on an LSM-800 confocal laser scanning microscope (Carl Zeiss Ltd., Cambridge, UK). Imaging settings were kept identical for all samples.

### Orthotopic Mouse Model of NCI-H358 Lung Cancers and Bioluminescence Imaging

To establish the orthotopic NCI-H358 lung cancer mouse model, approximately 5 × 10^5^ NCI-H358-Luc cells in 200 μL of PBS were injected into the 6-week-old female NOD SCID mice through the tail vein. Ten days after injection, for the detection of luciferase expression, NOD SCID mice were injected intraperitoneally with 150 mg/kg of body weight of d-Luciferin (Goldbio LUCK-1G, 200 μL of 15 mg/mL d-Luciferin in Dulbecco’s phosphate-buffered saline) and anesthetized with a mixture of oxygen/isoflurane. Bioluminescent signals were measured 10 min later with an IVIS Lumina II imaging system (Caliper Life Sciences, Inc., MA, USA), and total signal flux was quantified using Living Image Software (PerkinElmer) in units of photons/second. Throughout imaging, the animals were constantly supplied with 2% isoflurane gas anesthesia and placed on a thermostatically controlled heating pad (37 °C) to maintain body temperature. Based on the detected signals, mice with similar initial bioluminescence intensities were divided into four groups: pacDNA_LNA_ (inhalation), pacDNA_LNA_ (i.v. injection), scrambled pacDNA_LNA_ (inhalation), and PBS (inhalation). Each group was treated twice weekly for a total of 8 doses at a pacDNA_LNA_ dosage of 0.15 μmol/kg (3 nmol in 35 μL of PBS, ASO basis, inhalation), 0.5 μmol/kg (10 nmol in 200 μL of PBS, ASO basis, i.v. injection), or PBS only (35 μL, inhalation). Tumor growth of the four groups was measured through IVIS imaging once or twice per week for a total of 6 times with identical settings during day 10 to day 37. Animal body weights were measured weekly. On day 38, mouse lungs were collected for histological studies.

### Statistics

All of the data are presented as means ± SD unless otherwise specified. Statistical significance was assessed by using a two-tailed *t* test when only two groups were compared. For comparisons among more than two groups, evaluation of significance was performed using one-way ANOVA followed by Dunnett’s *post hoc* test. Figures were generated by using GraphPad Prism and Microsoft PowerPoint software.

## Supporting Information Available

Additional materials, instrumental and experimental details; scheme of synthesis of pacDNAs; characterization of brush polymer and pacDNAs; additional biodistribution data; plasma concentrations after inhalation; Western blot and viability analysis; additional confocal microscopy images; representative images of mouse lungs; additional KRAS IHC staining images; supplementary references (PDF)
