# Supplementary material for: Inhalable Bottlebrush Polymer Bioconjugates as Vectors for Efficient Pulmonary Delivery of Oligonucleotides
Source: ACS Nano. Author manuscript; Available in PMC 2025 Jan 9. (PMC10786149; doi:10.1021/acsnano.3c08660)
Supplement: Supplementary material [file NIHMS1955519-supplement-Supplementary_material.pdf]

Supporting Information

Inhalable bottlebrush polymer bioconjugates as vectors for efficient pulmonary delivery of oligonucleotides

*Yang Fang, Jiansong Cai, Mengqi Ren, Tongtong Zhong, Dali Wang, and Ke Zhang**

Department of Chemistry and Chemical Biology, Northeastern University, Boston, Massachusetts 02115, United States

*Corresponding author. Email: k.zhang@northeastern.edu

Supplementary Materials and Methods.

Materials and instrumentation. Phosphoramidites and supplies for oligonucleotide synthesis were purchased from Glen Research Co., USA. NCI-H358 and NCI-H1944 cell lines were purchased from American Type Culture Collection (Rockville, MD, USA). ω-Amine PEG methyl ether (Mn=10 kDa, PDI=1.05) was purchased from JenKem Technology, USA. All other common materials were obtained from Sigma-Aldrich Co. USA, Fisher Scientific Inc., USA, or VWR International LLC., USA, and were used as received unless otherwise indicated. For the purification of oligonucleotide, reverse-phase HPLC was performed on a Waters (Waters Co., MA, USA) Breeze 2 HPLC system coupled to a Symmetry® C18 3.5 µm, 4.6×75 mm reversed-phase column and a 2998 PDA detector, using TEAA buffer (0.1 M) and HPLC-grade acetonitrile as mobile phases. *N*,*N*-Dimethylformamide (DMF) GPC was carried out on a TOSOH EcoSEC HLC-8320 GPC system (Tokyo, Japan) equipped with a TSKgel Alpha-M, 7.8 mm ID × 30 cm column and RI/UV-Vis detectors. HPLC-grade DMF with 0.05 M LiBr was used as the mobile phase, and samples were run at a flow rate of 0.4 mL/min. Aqueous GPC measurements were carried out on a Waters Breeze 2 GPC system equipped with an Ultrahydrogel™ 1000, 7.8 × 30 cm column and three Ultrahydrogel™ 250, 7.8 × 30 cm columns and a 2998 PDA detector for the separation of as-synthesized polymers from monomers/oligonucleotides. MALDI-TOF MS measurements were performed on a Bruker Microflex LT mass spectrometer (Bruker Daltonics Inc., MA, USA).

Oligonucleotide synthesis. Oligonucleotides including modifications were synthesized on a Model 391 DNA synthesizer (Applied Biosystems, Inc., Foster City, CA) using standard solid-

phase phosphoramidite methodology. DNA strands were cleaved from the CPG support using aqueous ammonium hydroxide (28-30% NH_3 basis) at room temperature for 18 h. LNA-modified strands were synthesized on Universal Support III PS CPG, cleaved by treating with 2 M ammonia in methanol at RT for 60 min, and deprotected using aqueous ammonium hydroxide (28-30% NH_3 basis) at RT for 18 h. All strands were purified by reverse-phase HPLC liquid chromatography. The successful synthesis of all sequences was verified by MALDI-TOF MS.

Synthesis of diblock bottlebrush polymer. Two monomers, norbornenyl bromide (compound 1) and norbornenyl PEG (compound 2) were synthesized following previously published method .[1-4] Norbornenyl bromide (15 µmol, 5 equiv.) was dissolved in anhydrous toluene and placed in a Schlenk flask under N_2, which was then vacuumed and purged with N_2 gas three times. Then, a solution of Grubbs' catalyst 3rd generation (3 µmol, 1 equiv.) in deoxygenated toluene was rapidly added by gastight syringe. The reaction mixture was stirred for 30 min at RT followed by addition of a mixed solution (5 mL of toluene and 4.5 mL of dichloromethane) containing norbornenyl PEG (105 µmol, 35 equiv.) with a gastight syringe. The reaction mixture was further stirred for 2 h at RT. Thereafter, several drops of ethyl vinyl ether (EVE) were added into the Schlenk flask, and the reaction mixture was further stirred for 30 min. The mixture was then concentrated and precipitated (3×) into cold diethyl ether to give a white solid polymer (compound 3). The polymer was dried under high vacuum, redissolved in DMF, and mixed with an excess of sodium azide while stirring. After overnight reaction at RT, the materials were transferred to a dialysis tubing (MWCO, 6-8 kDa), dialyzed against Nanopure™ water for 24 h, and the dried by lyophilization to give azide-functionalized polymer (compound 4). DMF-GPC analysis determines the bottlebrush polymer with Mn =280 kDa, Mw =340 kDa, PDI ~1.2, yield

~93%. To quantify the number of azide groups per copolymer available for coupling, 10 nmol of the polymer was dissolved in 400 μL of DMF and conjugated with DBCO-modified Cyanine 5 (Lumiprobe, 1000 nmol). The reaction mixture was gently shaken on an Eppendorf Thermomixer at room temperature overnight. Thereafter, the solution was dialyzed against a Nanopure^{TM} water using dialysis tubing with a MWCO of 6-8 kDa for 72 h. The UV-Vis absorption of the polymer solution at 646 nm was measured and compared with a standard curve. The number of Cy5 molecules per polymer was calculated based on the known polymer concentration. Approximately 4.9 Cy5 tags were attached to each diblock brush copolymer (Figure S13).

Synthesis of pacDNAs. In a typical procedure, azide-functionalized bottlebrush polymer 4 (15 mg, 50 nmol) was dissolved in 800 μL of aqueous NaCl solution (2 M), to which DBCO-modified DNA or LNA (100 nmol) was added (dissolved in 200 μL of NaCl aqueous solution). The reaction mixtures were shaken gently for 17 h at 50 °C on an Eppendorf Thermomixer. Thereafter, the conjugation product was isolated using aqueous GPC. The conjugates were desalted using a NAP-25 column and lyophilized to yield a white powder (or blue powders for Cy5-labeled pacDNA).

Synthesis of PEG_{40k}-ASO. mPEG-Azide (MW 40k, PDI 1.02-1.05) was purchased from Creative PEGWorks, USA. mPEG-Azide (2mg, 50 nmol) was dissolved in 400 μL of aqueous NaCl solution (2 M), to which Cy5-labeled DBCO-modified LNA (60 nmol) was added (dissolved in 100 μL of NaCl aqueous solution). The reaction mixtures were shaken gently for 17 h at 50 °C on an Eppendorf Thermomixer. Thereafter, the conjugation product was isolated using

Fast Protein Liquid Chromatography (FPLC). The conjugates were desalted using a NAP-25 column and lyophilized to yield a blue powder.

Trapping efficiency of pacDNA and other controls in the agarose gel without CF-AM. A 0.28 w/v % agarose solution was prepared in hot Nanopure™ water. Vials with a diameter of 23 mm were filled with 1 mL of the agarose solution, hardened at room temperature, and stored at 4 °C until further use. A 200 μL of Cy5-labeled pacDNA solution (10 nmol dissolved in water, ASO basis), Cy5-labeled free ASO, Cy5-labeled PEG_{40k}-ASO, or Cy5-labeled free ASO mixed with Lipofectamine 3000 based on the standard protocol was then added onto the gel layer and incubated at 37 °C. After a 24 h incubation period, sample solutions were withdrawn, and the remaining agarose gels were rinsed with 1 mL of Nanopure™ water three times. The gels were subsequently melted at 60 °C, transferred to a 96-well black plate, and analyzed by measuring the fluorescence intensity of Cy5 using a BioTek® Synergy™ Neo2 Multi-Mode microplate reader (BioTek Inc., VT, USA). The amount of Cy5-labeled pacDNA and controls that reached the agarose gel were measured against standard curve generated using Cy5-labeled pacDNA, Cy5-labeled PEG_{40k}-ASO, or Cy5-labeled free ASO dissolved in an agarose gel solution (Figure S14A,B).

Synthesis of SSO 654-B peptide. DBCO-modified (100 nmol) SSO 654 and C-terminus azide-functionalized B-peptide (200 nmol, sequence: Ac-RXRRBRRXRRBRXBK(N_3), 6-aminohexanoic acid (X), β-alanine (B)) were dissolved in 1500 μL of aqueous NaCl solution (2 M). The reaction mixture was shaken gently for 17 h at 50 °C on an Eppendorf Thermomixer. Thereafter, the conjugation product was purified by reverse-phase HPLC liquid chromatography,

and the successful synthesis of conjugate was confirmed by LC-ESI-MS (Calculated: 8897 Da; Measured 8897.97 Da, Figure S15A,B). Purified SSO 654-B peptide conjugate was desalted with a NAP-25 column, stored as lyophilized powder, and kept at -20 °C.

Cell culture. NCI-H358 cells were transfected with luciferase (NCI-H358-luc), thus the cell growth could be monitored based on the detection of bioluminescence signals.[5] Cells were in cultured in RPMI 1640 supplied with 10% fetal bovine serum (FBS), 100 units/mL penicillin, and 100 µg/mL streptomycin at 37 °C in a humidified atmosphere containing 5% CO_2. The culture medium was changed every two days. The cells were harvested with 0.25% trypsin and 2.21mM EDTA through trypsinization.

Western blot analysis of NCI-H358-luc cell line. The gene regulation efficacy of $pacDNA_{LNA}$ KRAS and $pacDNA_{PO}$ KRAS *in vitro* was evaluated in cell line using western blot. NCI-H358-luc cells were seeded into 24-well plates at 1.5×10^5 cells per well in 1mL of full-growth medium and cultured overnight at 37 °C with 5% CO_2. Cells were incubated with $pacDNA_{PO}$ KRAS, scrambled $pacDNA_{PO}$ control, $pacDNA_{LNA}$ KRAS, and scrambled $pacDNA_{LNA}$ control (5 and 10 µM, ASO basis) in 500 µL of serum-free medium for 6 h. Thereafter, 500 µL of full-growth medium was added into the wells and cells were further cultured for another 66 h. Whole cell lysate was collected in 60 µL of radioimmunoprecipitation assay cell lysis buffer (RIPA) containing Halt™ protease and phosphatase inhibitor cocktail, and 5 mM of EDTA (ThermoFisher, MA, USA). Protein concentrations were measured by a BCA protein assay with BSA as the protein standard. Equal amounts (20 µg/lane) of protein samples were separated on 4-20% gradient SDS-PAGE, and electro-transferred to nitrocellulose membrane. The membranes

were blocked for 1 h at RT with 3% bovine serum albumin (BSA) in tris-buffered saline supplemented with 0.05% Tween 20, then incubated with primary antibodies against vinculin (1:2000 dilution, Sigma Aldrich, MO, USA) and KRAS (1:2000 dilution, Novus Biologicals, CO, USA) at 4°C overnight. After washing and incubation with anti-mouse secondary antibody (1:5000 dilution, Cell Signaling Technology, MA, USA) at RT for 1 h, protein bands were visualized by using an ECL Western Blotting Substrate (Pierce, ThermoFisher, MA, USA).

MTT assay with NCI-H358-luc cell line. The cytotoxicity of brush polymer, pacDNA$_{LNA}$ KRAS, and scrambled pacDNA$_{LNA}$ were evaluated with the MTT assay against NCI-H358-luc cell. Briefly, NCI-H358-luc cells were seeded into 96-well plates at 8000 cells per well in 200 μL medium and cultured overnight. The cells were then treated with brush polymer (0.5 to 5 μM), pacDNA$_{LNA}$ KRAS (0.5 to 10 μM, ASO basis), and scrambled pacDNA$_{LNA}$ (0.5 to 10 μM, ASO basis). Cells treated with PBS buffer were used as a negative control. After 72 h of incubation, 10 μL of 5 mg/mL MTT stock solution in PBS was added to each well. The cells were incubated for another 3 h, and the medium containing unreacted MTT was removed carefully. The resulting blue formazan crystals were dissolved in 200 μL per well DMSO, and the absorbances (560 nm) were measured on a BioTek® Synergy™ Neo2 Multi-Mode microplate reader (BioTek Inc., VT, USA).

Plasma pharmacokinetics (PK). Immunocompetent C57BL/6 mice were utilized to investigate the plasma pharmacokinetics (PK) of inhaled pacDNA and free LNA. For administration, Cy5-labeled pacDNA or free LNA (5 nmol dissolved in 50 µL of PBS, ASO basis) was intratracheally delivered to the mice using a microsprayer device (PenWu Device for Mouse, Bio

Jane Trading Limited, Shanghai, China). Blood samples (25 µL) were collected from the submandibular vein at various time points following inhalation of the Cy5-labeled samples (1 h, 4 h, 24 h, 48 h, 72 h, 14 d). BD Vacutainer blood collection tubes with lithium heparin were used for sample collection. Heparinized plasma was obtained by centrifuging the samples at 3,000 rpm for 15 minutes. The plasma was then aliquoted into a 96-well plate, and the fluorescence intensity was measured using a BioTek Synergy HT plate reader (BioTek Instruments Inc.). To estimate the amounts of ASO present in the blood samples, standard curves were established for each sample. Samples with known quantities of ASO were incubated with freshly collected plasma for 1 hour at room temperature, after which the fluorescence intensity was measured. The established standard curves were then used to determine the concentrations of ASO in the blood samples collected from the mice at different time points.

EGFP-654 animal treatment. 6-week-old female EGFP-654 mice were divided into six groups: pacDNA-654 (inhalation), pacDNA-654 (i.v. injection), SSO 654-B peptide (i.v. injection), scrambled pacDNA-654 (inhalation), brush polymer (inhalation), and PBS (inhalation). Animals were treated once a day for consecutive four days for a total of four doses at dosage of 0.15 µmol/kg for pacDNA-654 and scrambled pacDNA-654 (3 nmol in 35 µL PBS, DNA basis, inhalation), 0.075 µmol/kg for brush polymer (1.5 nmol in 35 µL PBS, equal to amount of brush polymer of inhaled pacDNA groups, inhalation), 1.0 µmol/kg for pacDNA-654 and SSO 654-B peptide (20 nmol in 200 µL PBS, DNA basis, iv injection), or PBS only (35 µL, inhalation). Animals were sacrificed by CO_2 inhalation at one week post last administration, and lung tissues were collected and immediately frozen for RNA isolation.

Histology analysis. Orthotopic lung tumor mouse lung tissue samples from pacDNA$_{LNA}$ KRAS (inhalation)-treated group and control groups were collected, fixed with 10% formalin neutral buffer solution, and embedded in paraffin. Immunohistochemistry study using mouse anti-KRAS primary antibody (1:1000 dilution, Novus Biologicals, CO, USA) and goat anti-mouse secondary antibody (1:5000 dilution, ThermoFisher, MA, USA), and H&E staining study were carried out at iHisto Inc. according to standard protocol.

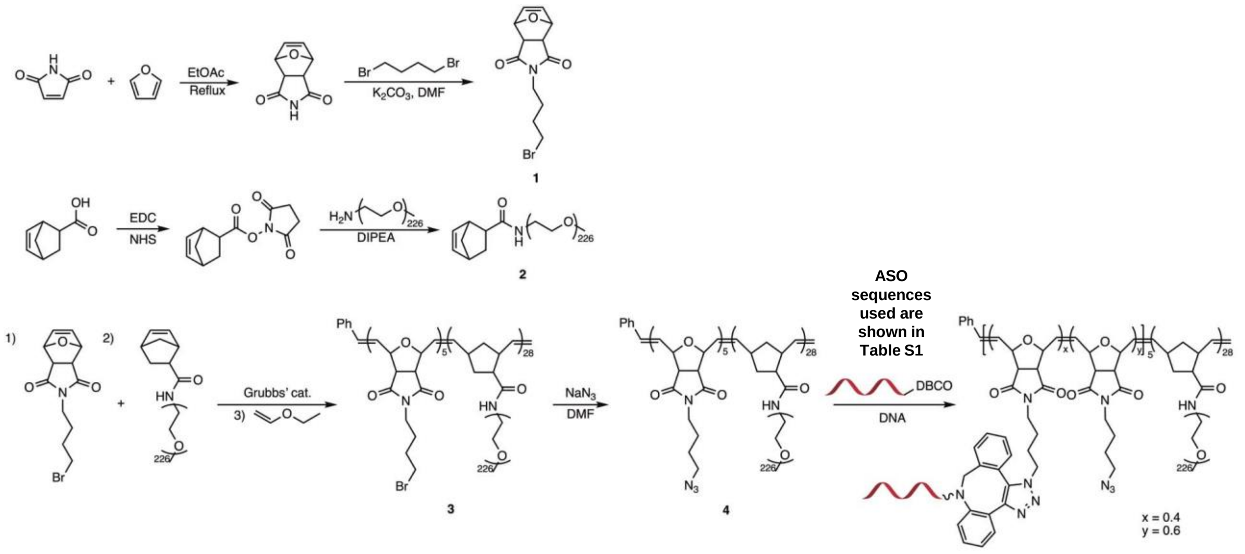

Scheme S1. Synthesis of pacDNA$_{LNA}$ KRAS, pacDNA$_{LNA}$ Scr, Cy5-labeled pacDNA$_{LNA}$ KRAS, pacDNA$_{PO}$ KRAS, pacDNA$_{PO}$ Scr, pacDNA-654, and pacDNA-654 Scr.

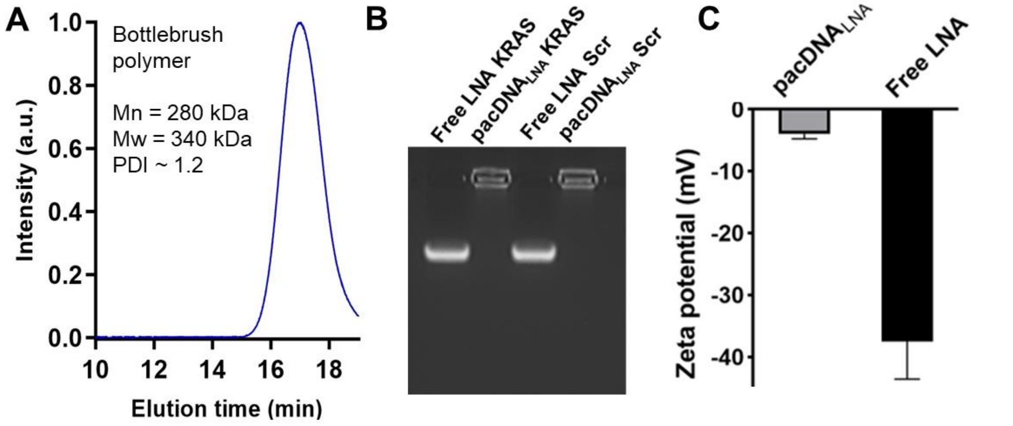

Figure S1. **Characterization of bottlebrush polymer and pacDNA$_{LNA}$s.** A) DMF GPC chromatogram of the bottlebrush polymer. B) Agarose gel electrophoresis (2%) of purified pacDNA$_{LNA}$s, and free LNAs. C) Zeta potential measurements of free LNA and pacDNA$_{LNA}$ in Nanopure^{TM} water (pH ~ 6.40).

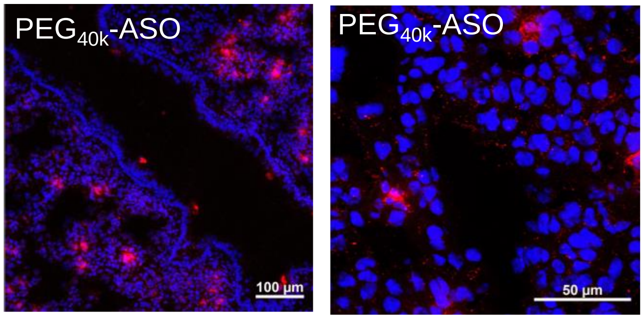

Figure S2. The distribution of PEG_{40k}-ASO in normal mouse lung (C57BL/6).

Representative images of PEG_{40k}-ASO distribution in mouse lung airway and parenchyma 24 h post inhalation.

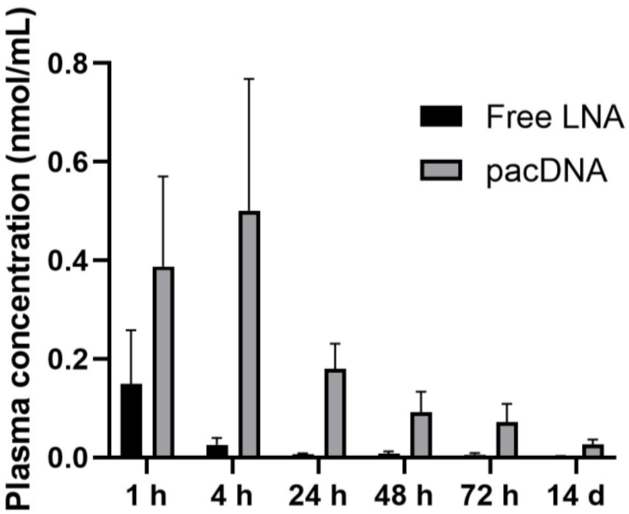

Figure S3. **Plasma concentrations of pacDNA or free LNA (ASO basis) at various time points after inhalation in C57BL/6 mice.**

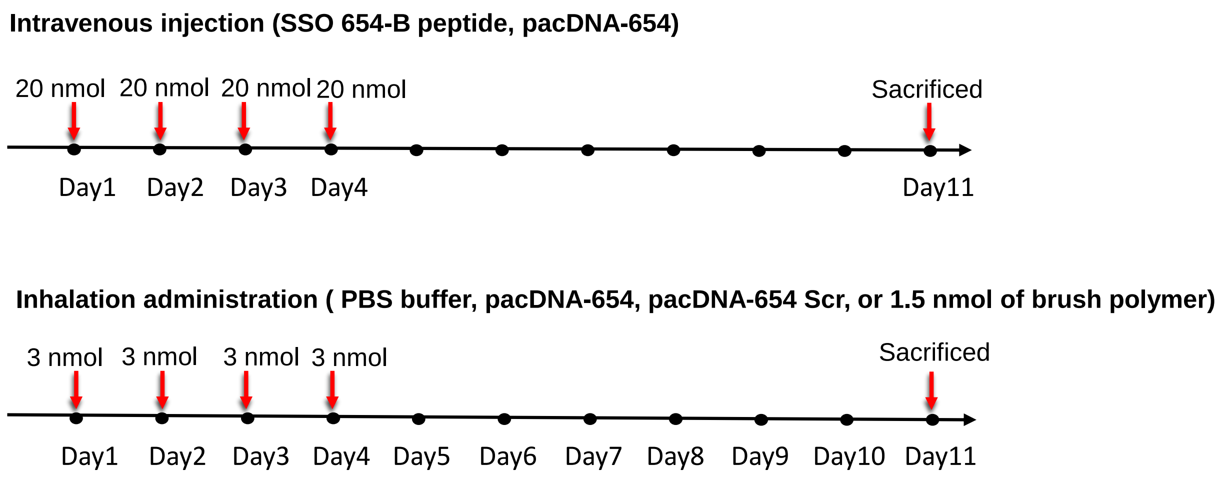

Figure S4. Schedule of EGFP-654 mice treatment with pacDNA-654 at dosage of 0.15 µmol/kg (inhalation) or 1.0 µmol/kg (iv injection), pacDNA-654 Scr at dosage of 0.15 µmol/kg (inhalation), brush polymer at dosage of 0.075 µmol/kg (inhalation), SSO 654-B peptide at dosage of 1.0 µmol/kg (iv injection), and PBS (inhalation).

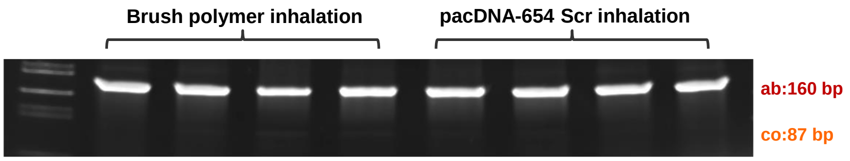

Figure S5. RT-PCR of EGFP RNA from lung tissue of EGFP-654 mice. Mice were treated with inhaled brush polymer or pacDNA-654 Scr.

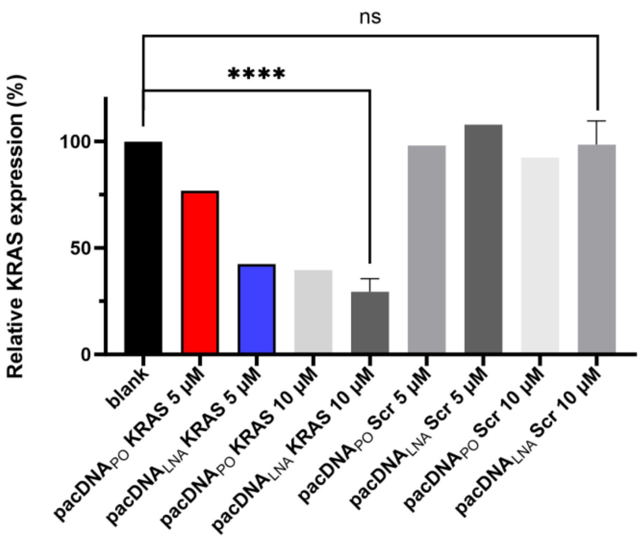

Figure S6. Western blot analysis of KRAS protein expression in NCI-H358-Luc cells after treatment with pacDNA and controls. ****p<0.0001, two-tailed test.

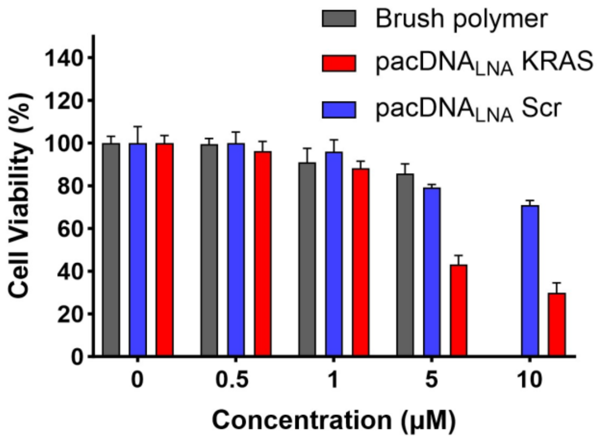

Figure S7. Viability of NCI-H358-Luc cells treated with brush polymer, pacDNA$_{LNA}$ KRAS, and scrambled pacDNA$_{LNA}$, as determined by an MTT cell viability assay.

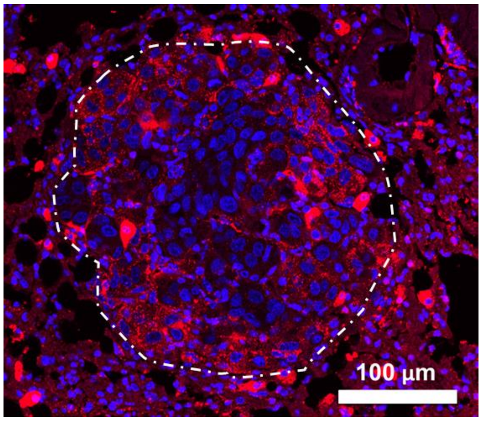

Figure S8. **Representative confocal image of Cy5-labeled pacDNA$_{LNA}$ (red) distribution in NOD SCID mouse lung bearing NCI-H358 human lung cancer at 24 h post inhalation delivery.** Cell nuclei are stained with Hoechst 33342 (blue), tumor cells were circled by white dashed line.

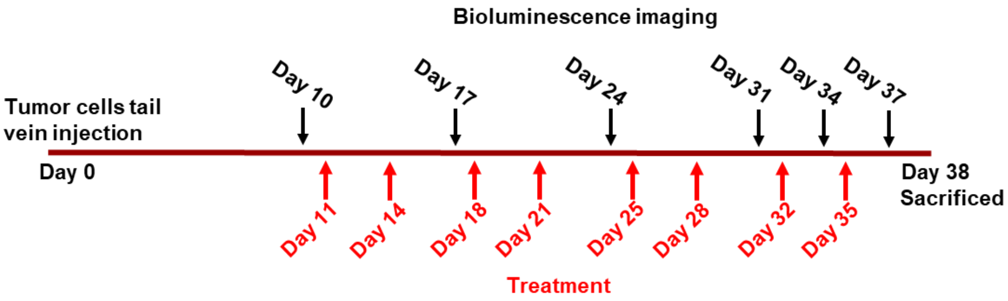

Figure S9. Schedule of NCI-H358-Luc tumor inoculation, IVIS bioluminescence imaging, and treatment with pacDNA$_{LNA}$ KRAS at dosage of 0.15 µmol/kg (inhalation) or 0.5 µmol/kg (iv injection).

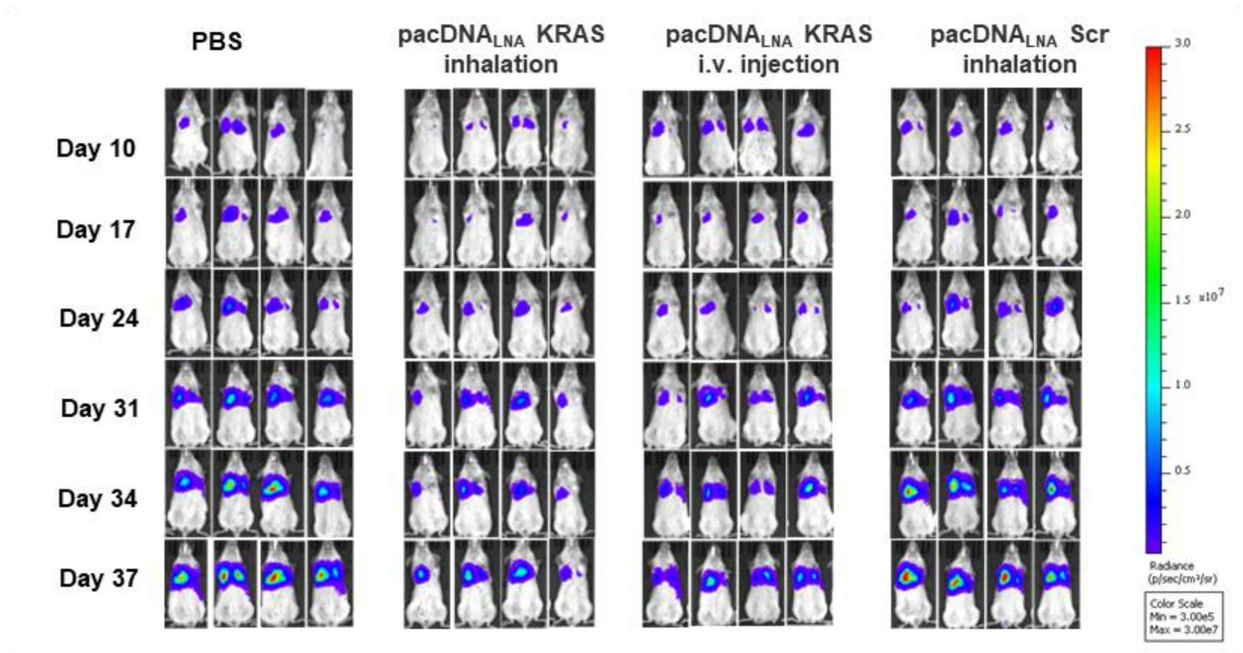

Figure S10. Representative NCI-H358-luc luciferase mediated bioluminescence intensities in mice of different groups. A) PBS-treated, B) inhaled pacDNA$_{LNA}$ KRAS-treated, C) i.v. injected pacDNA$_{LNA}$ KRAS-treated, and D) inhaled pacDNA$_{LNA}$ Scr-treated group.

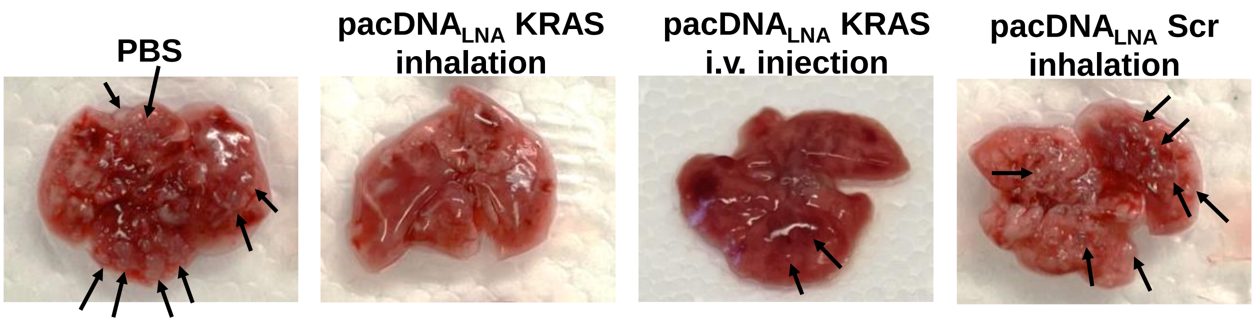

Figure S11. Representative images of collected mouse lung at day 38 from different groups. Surface nodules on the lung are marked by black arrows.

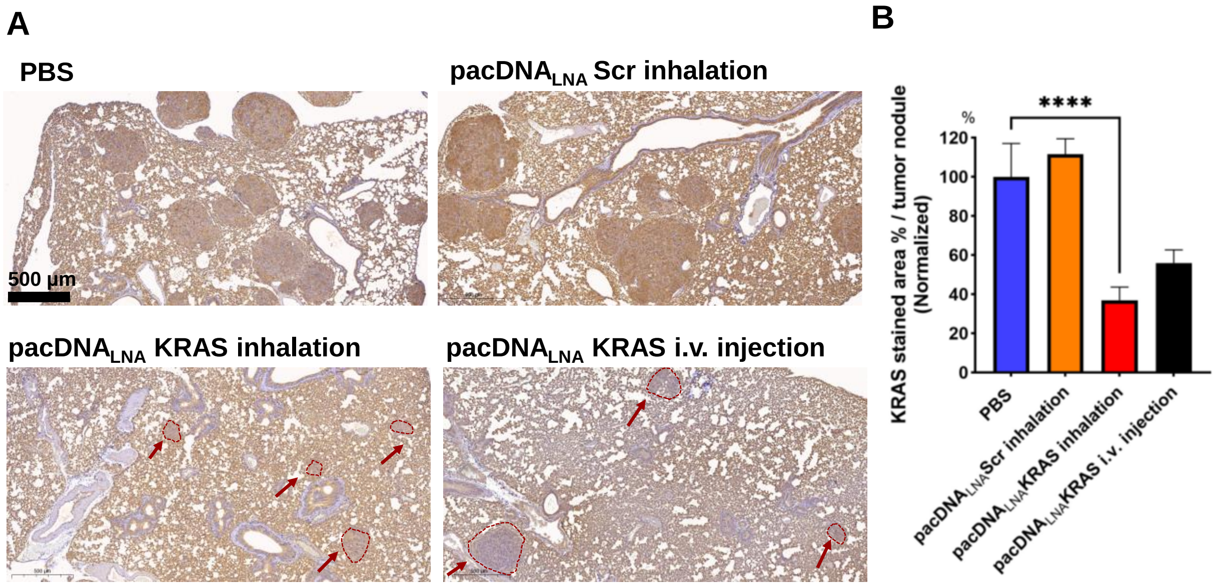

Figure S12. KRAS IHC staining and quantification. A) Representative immunohistochemical staining for KRAS in day 38- harvest lung tissues, showing reduced KRAS expression of nodules in inhaled pacDNA$_{LNA}$ KRAS-treated groups vs controls. Scale bar: 500 µm. Surface nodules are marked by red dashed lines. B) Relative KRAS stained area per tumor nodule of mouse lungs (quantified with ImageJ) as determined by immunohistostaining. Normalized to PBS-treated group. **** $p < 0.0001$ (two-tailed test).

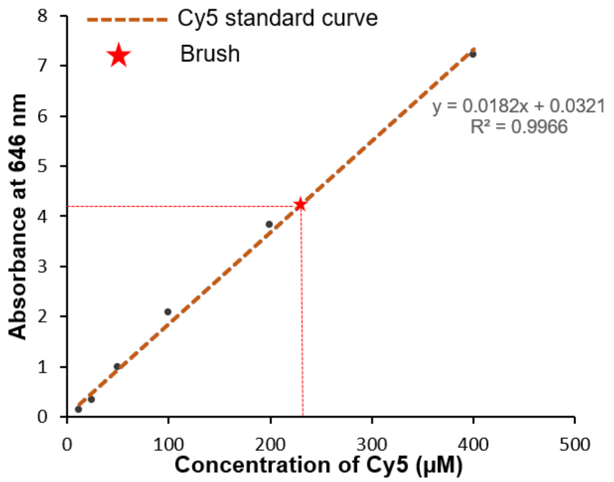

Figure S13. Standard curve generated using free Cyanine 5 in Nanopure™ water for the quantification of available azide groups on azide-functionalized brush polymer.

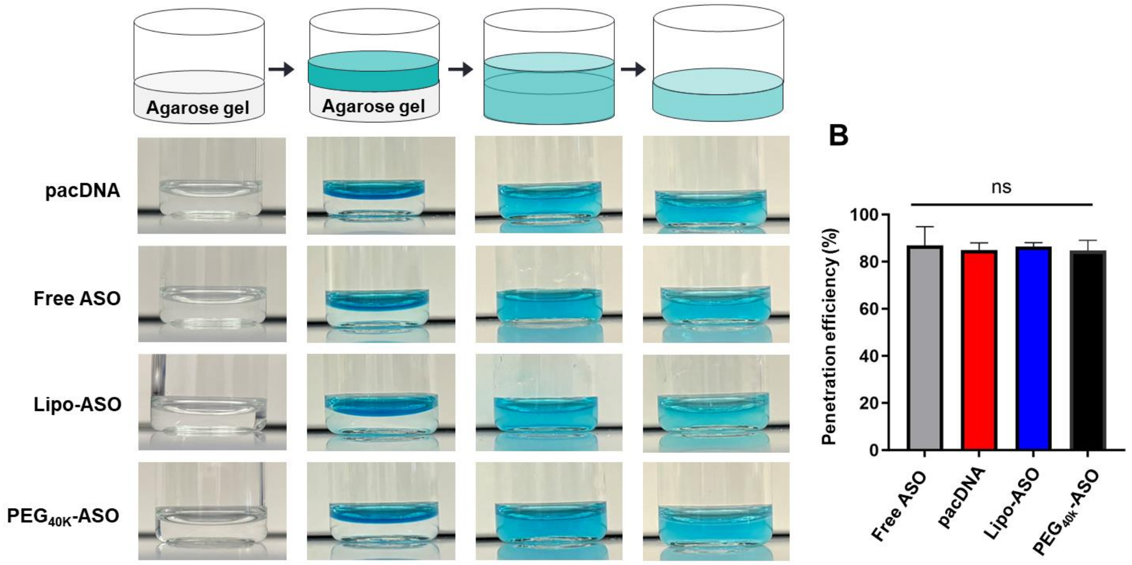

Figure S14. **Trapping efficiency of pacDNA and other controls in the agarose gel without CF-AM.** A) Cy5-labeled samples were added to the top of the gel, both pacDNA and other control groups showed similar gel trapping efficiency after 24 h of incubation at 37°C. B) Extent of gel trapping after 24 h of incubation at 37°C.

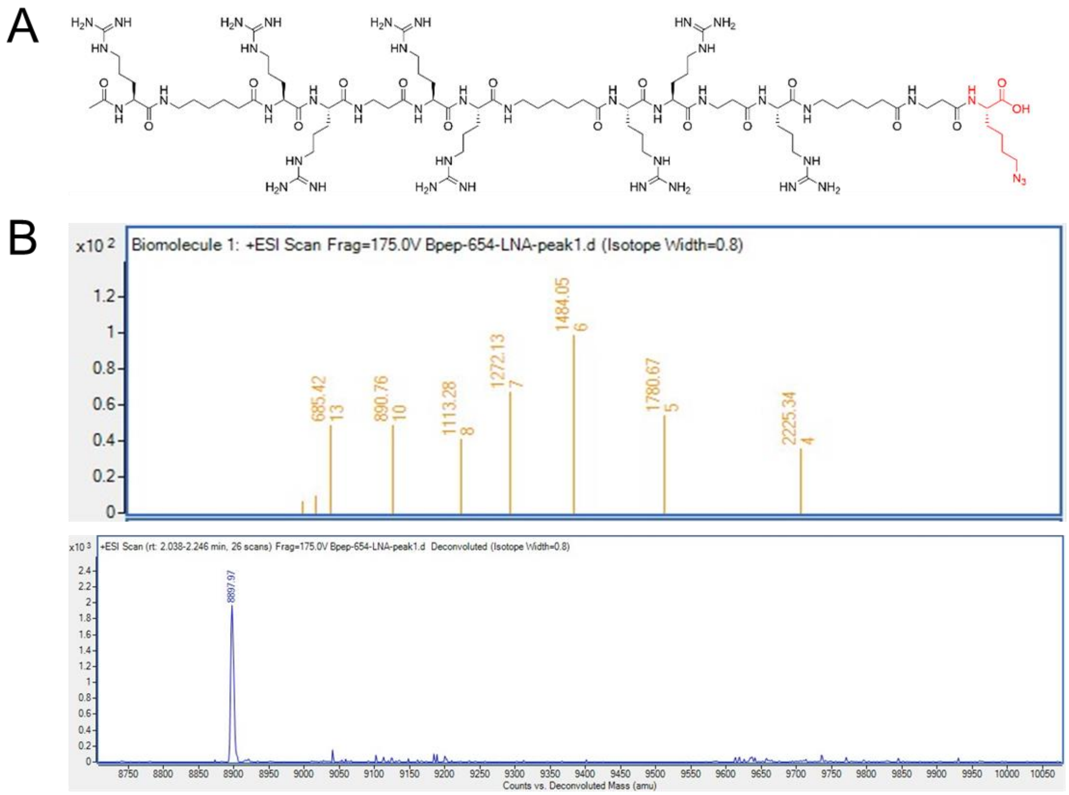

Figure S15. B peptide and SSO 654-B peptide conjugate. A) Structure of azide-modified B peptide. B) Mass spectrum of SSO 654-B peptide conjugate.

Table S1. ASO sequences used in this study.

Name	Sequence
1. Cy5-labeled antisense LNA KRAS	5'- **GCTATTAGGAGTCTTT**-Cy5-3'
2. DBCO-modified antisense LNA KRAS	5'-DBCO-**GCTATTAGGAGTCTTT**-3'
3.Cy5-labeled and DBCO-modified antisense LNA KRAS	5'-DBCO-**GCTATTAGGAGTCTTT**-Cy5-3'
4. DBCO-modified scramble LNA KRAS	5'-DBCO-**ATGTCCGTTGTGTATA**-3'
5. DBCO-modified antisense DNA KRAS	5'-DBCO-GCTATTAGGAGTCTTT-3'
6. DBCO-modified scramble DNA KRAS	5'-DBCO-ATGTCCGTTGTGTATA-3'
7. DBCO-modified SSO 654	5'-DBCO-**GCTATTACCTTAACCCAG**-3'
8. DBCO-modified scramble SSO 654	5'-DBCO-**GCAAATTCCTATTCCCAG**-3'

Upper case: PO DNA. **Bold**: locked nucleic acid (LNA) modification.

References:

1. Lu, X.; Jia, F.; Tan, X.; Wang, D.; Cao, X.; Zheng, J.; Zhang, K., Effective Antisense Gene Regulation via Noncationic, Polyethylene Glycol Brushes. *J. Am. Chem. Soc.* **2016,** *138* (29), 9097-9100.

2. Pontrello, J. K.; Allen, M. J.; Underbakke, E. S.; Kiessling, L. L., Solid-Phase Synthesis of Polymers Using the Ring-Opening Metathesis Polymerization. *J. Am. Chem. Soc.* **2005,** *127* (42), 14536-14537.

3. Fang, Y.; Lu, X.; Wang, D.; Cai, J.; Wang, Y.; Chen, P.; Ren, M.; Lu, H.; Union, J.; Zhang, L.; Sun, Y.; Jia, F.; Kang, X.; Tan, X.; Zhang, K., Spherical Nucleic Acids for Topical Treatment of Hyperpigmentation. *J. Am. Chem. Soc.* **2021,** *143* (3), 1296-1300.

4. Chen, P.; Wang, D.; Wang, Y.; Zhang, L.; Wang, Q.; Liu, L.; Li, J.; Sun, X.; Ren, M.; Wang, R.; Fang, Y.; Zhao, J. J.; Zhang, K., Maximizing TLR9 Activation in Cancer Immunotherapy with Dual-Adjuvanted Spherical Nucleic Acids. *Nano Lett.* **2022,** *22* (10), 4058-4066.

5. Mu, Q.; Yu, J.; Griffin, J. I.; Wu, Y.; Zhu, L.; McConnachie, L. A.; Ho, R. J. Y., Novel drug combination nanoparticles exhibit enhanced plasma exposure and dose-responsive effects on eliminating breast cancer lung metastasis. *PLOS ONE* **2020,** *15* (3), e0228557.
